# Neural activity during interoceptive awareness and its associations with alexithymia—An fMRI study in major depressive disorder and non-psychiatric controls

**DOI:** 10.3389/fpsyg.2015.00589

**Published:** 2015-05-27

**Authors:** Christine Wiebking, Georg Northoff

**Affiliations:** ^1^Cluster of Excellence in Cognitive Sciences, Department of Sociology of Physical Activity and Health, University of PotsdamPotsdam, Germany; ^2^Mind, Brain Imaging and Neuroethics, Institute of Mental Health Research, University of OttawaOttawa, ON, Canada; ^3^Graduate Institute of Humanities in Medicine, Taipei Medical UniversityTaipei, Taiwan; ^4^Taipei Medical University-Shuang Ho Hospital, Brain and Consciousness Research CenterNew Taipei City, Taiwan; ^5^Department of Psychology, National Chengchi UniversityTaipei, Taiwan; ^6^Center for Cognition and Brain Disorders, Normal University HangzhouHangzhou, China

**Keywords:** major depressive disorder, alexithymia, interoceptive awareness, insula, sACC, interoception, fMRI, neuroimaging

## Abstract

**Objective:** Alexithymia relates to difficulties recognizing and describing emotions. It has been linked to subjectively increased interoceptive awareness (IA) and to psychiatric illnesses such as major depressive disorder (MDD) and somatization. MDD in turn is characterized by aberrant emotion processing and IA on the subjective as well as on the neural level. However, a link between neural activity in response to IA and alexithymic traits in health and depression remains unclear.

**Methods:** A well-established fMRI task was used to investigate neural activity during IA (heartbeat counting) and exteroceptive awareness (tone counting) in non-psychiatric controls (NC) and MDD. Firstly, comparing MDD and NC, a linear relationship between IA-related activity and scores of the Toronto Alexithymia Scale (TAS) was investigated through whole-brain regression. Secondly, NC were divided by median-split of TAS scores into groups showing low (NC-low) or high (NC-high) alexithymia. MDD and NC-high showed equally high TAS scores. Subsequently, IA-related neural activity was compared on a whole-brain level between the three independent samples (MDD, NC-low, NC-high).

**Results:** Whole-brain regressions between MDD and NC revealed neural differences during IA as a function of TAS-DD (subscale difficulty describing feelings) in the supragenual anterior cingulate cortex (sACC; BA 24/32), which were due to negative associations between TAS-DD and IA-related activity in NC. Contrasting NC subgroups after median-split on a whole-brain level, high TAS scores were associated with decreased neural activity during IA in the sACC and increased insula activity. Though having equally high alexithymia scores, NC-high showed increased insula activity during IA compared to MDD, whilst both groups showed decreased activity in the sACC.

**Conclusions:** Within the context of decreased sACC activity during IA in alexithymia (NC-high and MDD), increased insula activity might mirror a compensatory mechanism in NC-high, which is disrupted in MDD.

## Introduction

Alexithymia is a multifaceted personality construct characterized by individuals having difficulties identifying or describing feelings. The term literally means “no words for feelings” and was introduced by Sifneos (1973) based on observations of psychosomatic disorder patients. Subsequent investigations have identified a link between alexithymic traits and other mental illnesses, including affective disorders like major depression (Bankier et al., 2001; Honkalampi et al., 2001; Saarijärvi et al., 2001; Leweke et al., 2012) and somatoform disorder (Karvonen et al., 2005; Burba et al., 2006). The 20-item Toronto Alexithymia Scale (TAS-20, Bagby et al., 1994a,b; see De Gucht and Heiser, 2003 for review) is the most commonly used self-report measurement of alexithymia. In line with Sifneos' observations, TAS scores were found to be associated with increased levels of subjective body awareness (Nakao et al., 2002; Kano et al., 2007; Ernst et al., 2014) and depressive symptoms (Honkalampi et al., 2001; Saarijärvi et al., 2001). The described triangular interconnection between alexithymia, bodily awareness and depressive symptoms (see Harshaw, 2015 for review) is also evident in the general population (see Honkalampi et al., 2000 on depression and alexithymia; see Mattila et al., 2008 on alexithymia and somatization). This supports the view of alexithymia being a relatively stable personality trait (Luminet et al., 2001; Saarijarvi et al., 2006; Stingl et al., 2008) and emphasizes the need for more investigations in non-psychiatric participants. Despite these strong relationships between alexithymic traits and major depression, both of which include aberrant emotional and body awareness, the association between neural activity in response to body awareness and alexithymic traits in health and depression remains to be described.

A growing body of research in the field of neuroimaging has investigated body-related processes in the form of interoceptive stimuli. Neural activity during interoceptive awareness (IA)–the awareness of stimuli, such as the heartbeat, that originate within the body–is considered an important factor in the processing of emotions (Damasio, 1999; Lamm and Decety, 2008; Lamm and Singer, 2010; Gu et al., 2013). Alexithymia and IA are therefore linked by a similar feature, namely the perception of body-related and emotional stimuli. As proposed previously (e.g., by Gu et al., 2013; Kano and Fukudo, 2013), alexithymia might show differential associations with IA-related neural activity in brain regions implicated in subjective interoceptive and emotional experience. However, few neuroimaging studies have investigated neural activity during IA in affective disorders like major depressive disorder (MDD). These have shown aberrant neural activity during IA in MDD, particularly decreased neural activity in the insula (Wiebking et al., 2010, 2015; Avery et al., 2014). Although no investigations have directly targeted neural processes during IA in alexithymia, neuroimaging studies have shown altered neural activity in response to emotional stimuli in brain structures associated with IA such as the insula and the anterior cingulate cortex (ACC) (see Wingbermühle et al., 2012 for review). For example, Karlsson et al. (2008) used visual emotional stimuli in H^15^_2_O-PET (positron emission tomography) to investigate regional cerebral blood flow changes in healthy participants, which were recruited on the basis of their high and low TAS scores. During emotional processing, high-alexithymic individuals showed increased cerebral blood flow in, amongst other brain regions, the insula. Less activation was reported in the anterior cingulate when comparing high vs. low alexithymic individuals. In another PET study comparing high and low non-psychiatric TAS scorers, Kano et al. (2007) investigated the effects of visceral stimulation. Alexithymic participants showed increased activity during colonic distension in the insula, but increased activity in the anterior cingulate during physical stimulation. In addition to PET, a growing number of functional magnetic resonance imaging (fMRI) studies have found that alexithymia is associated with aberrant task-evoked neural activity in response to emotional stimuli in the insula and ACC. In this context, increased neural activity in the insula, particularly in response to bodily stimuli, might be associated with hyperawareness of somatosensory signals in alexithymia, which has been described on the level of subjective emotional experience and behavior (e.g., De Gucht and Heiser, 2003; Nakao and Barsky, 2007; Wingbermühle et al., 2012; Kano and Fukudo, 2013).

The observation of enhanced insula activity seems to be a more consistent characteristic in alexithymia research compared to decreased ACC activity. Increased insula activity is frequently observed in response to awareness of interoceptive stimuli such as the heartbeat, breathing or bladder state (Simmons et al., 2006; Pollatos et al., 2007; Farb et al., 2013; Wiebking et al., 2014, 2015). In alexithymia, increased insula activation was shown in response to a variety of emotional tasks, such as viewing emotional pictures (negative or positive) (Deng et al., 2013), viewing facial expressions (happy or sad) (Lemche et al., 2013), viewing painful pictures (human hands and feet) (Moriguchi et al., 2007), a trauma script imagery task (Frewen et al., 2008) (posterior insula), or in response to an empathy for pain paradigm (Bird et al., 2010). However, simultaneously observed neural activity in regions of the ACC showed inconsistent patterns across these fMRI studies, ranging from increased activity (Deng et al., 2013; Lemche et al., 2013; see also Heinzel et al., 2010) to decreased responses (Moriguchi et al., 2007; Frewen et al., 2008; see also Berthoz et al., 2002; Leweke et al., 2004). Combining self-reported measures of body awareness and alexithymia with biochemical measurements, a recent MRS (magnetic resonance spectroscopy) study investigated the association between alexithymia (TAS), body awareness (Porges, 1993) and metabolite concentrations of GABA (gamma-aminobutyric acid) and glutamate in the insula and ACC (Ernst et al., 2014). Levels of glutamate, the primary excitatory neurotransmitter in the brain, were positively related to alexithymia and body awareness in the insula. The finding of increased glutamate-mediated excitatory transmission within the insula of alexithymic individuals supports the aforementioned studies of increased insula activity, although no direct neuro-biochemical relationship was demonstrated. Considering the methodological and interpretative shortcomings of MRS (Duncan et al., 2013), the results are further suggestive of an aberrant interoceptive signal-to-noise ratio in the insula in alexithymia. On the other hand, levels of GABA, the primary inhibitory neurotransmitter in the brain, were positively related to alexithymia in the ACC. Mediating a reduction of neural responses, an enhanced GABAergic transmission in the ACC may account for the decreased neural activity in alexithymia in this region (Ernst et al., 2014).

Despite the fact that certain brain regions (insula, ACC) and stimuli types (body-related interoceptive and emotional stimuli) play a role in alexithymia (see for reviews Wingbermühle et al., 2012; Moriguchi and Komaki, 2013; van der Velde et al., 2013), direct associations between alexithymic traits and neural activity during IA remain unclear. To clarify these questions, a well-established paradigm was used in the current fMRI study to investigate neural activity during internal (heartbeat counting) and external awareness (tone counting) in MDD and non-psychiatric controls (NC) (Wiebking et al., 2014, 2010, 2015). The 20-item TAS was used to objectify alexithymia. It is the most widely used self-report instrument to measure the degree of alexithymia and consist of three subscales: difficulties identifying feelings (DI), difficulties describing feelings (DD) and external oriented thinking (EO) (Bagby et al., 1994a,b). In the first step, a linear relationship between IA-related neural activity and TAS scores comparing MDD and NC was investigated on a whole-brain level, where TAS-DD showed significant effects. In the second step, the self-reported scores of alexithymia in the NC group, as assessed by TAS-DD, were dichotomized using a median-split. This led to two NC subgroups, those with high and those with low alexithymia scores. MDD and NC having high TAS-DD scores showed equally high alexithymia scores. Differences in fMRI response to IA between the three groups–high alexithymic NC, low alexithymic NC, and MDD–were then examined on the whole-brain level.

Based on previous neuroimaging findings in studies investigating neural activation of alexithymia or IA, it was hypothesized that differences would occur specifically during IA in the insula and medial-frontal cortex depending on alexithymia status. Though the literature depicts less consistent findings regarding medial prefrontal activity compared to insula responses in alexithymia, non-psychiatric alexithymic individuals were expected to show reduced neural activity during IA in the medial prefrontal regions, such as the ACC. This assumption was based on decreased task-evoked activity in the ACC in association with alexithymia (e.g., Berthoz et al., 2002; Leweke et al., 2004; Moriguchi et al., 2007; Frewen et al., 2008; Karlsson et al., 2008). Moreover, lower connectivity within medial frontal areas of the default mode network in alexithymia (Liemburg et al., 2012), lower ACC gray matter volumes in alexithymia (Borsci et al., 2009; Grabe et al., 2014) and a positive connection between alexithymia and GABA in the ACC (Ernst et al., 2014) support the assumption of decreased neural responses in alexithymia. According to consistent findings of increased insula activity in response to emotional and bodily stimuli in alexithymia (e.g., Moriguchi et al., 2007; Frewen et al., 2008; Deng et al., 2013; Lemche et al., 2013; see Wingbermühle et al., 2012; Kano and Fukudo, 2013 for reviews), increased neural activity during IA was expected in the insula in non-psychiatric alexithymic participants. In line with previous neuroimaging studies investigating neural activity during IA in MDD (Wiebking et al., 2010, 2015; Avery et al., 2014), the alexithymic MDD group was hypothesized to show reduced neural activity in the insula.

## Methods

### Participants

A group of 22 patients suffering from MDD and a group of 30 non-psychiatric controls (NC) underwent fMRI scanning. All NC completed self-report measurements of the TAS as well as fMRI scanning, but TAS scores for 6 MDD patients were missing. Hence, 30 NC (mean age 33.73 ± 11.62 years, range 22–60 years; mean years of education: 16.05 ± 2.42; 15 female participants) and 16 MDD patients (mean age 41.19 ± 11.78 years, range 23–58 years; mean years of education: 15.72 ± 2.88; 11 female participants) completed both study parts and were included in further analysis.

Patients with MDD were recruited in an acute state from the Department of Psychiatry (University of Magdeburg) or from the state hospital of Uchtspringe. Eligibility screening procedures included the 21-item Beck Depression Inventory (BDI, Beck et al., 1961) (*n* = 14 MDD: 30.57 ± 7.00; NC: n.a.) and the 20-item Beck Hopelessness Scale (BHS, Beck et al., 1974) (*n* = 14 MDD: 12.29 ± 4.60; NC: 4.60 ± 3.91) (please also refer to Supplementary Table 1). Diagnosis of MDD was made by the participants' treating psychiatrists according to DSM-IV standards (Diagnostic and Statistical Manual of Mental Disorders, 4th edition; American Psychiatric Association, 1994). Exclusion criteria included major medical illnesses, histories of seizures, metallic implants, a history of substance dependence, head trauma with loss of consciousness, pregnancy, and criteria for any psychiatric disorder other than MDD. NC were recruited from the local community and were questioned about psychiatric, neurological, or medical diseases using a custom-made semistructured clinical questionnaire. All participating individuals gave their written informed consent before participating in this study. The study was approved by the local ethics committee.

### Psychometric measures

The 20-item Toronto Alexithymia Scale (TAS-20, Bagby et al., 1994a) is the most widely used and validated self-report measure of alexithymia. The factors of the TAS-20 are replicable across cultures (Taylor et al., 2003) and the current study used the validated German version of the TAS-20 (Bach et al., 1996). Items are rated using a 5-point Likert scale, ranging from 1 (strongly disagree) to 5 (strongly agree). The 20-items are categorized in three dimensions: difficulties identifying feelings (TAS-DI, example item: “I am often confused about what emotion I am feeling”), difficulties describing feelings (TAS-DD, example item: “It is difficult for me to find the right words for my feelings”) and externally oriented thinking (TAS-EO, example item: “I prefer talking to people about their daily activities rather than their feelings.”). High TAS scores indicate high alexithymic traits, i.e., more difficulties describing or identifying feelings.

### fMRI paradigm

A well-established fMRI design for investigating interoceptive and exteroceptive awareness was used in this study (Supplementary Figure 2B). The basic concept of the paradigm was introduced by Critchley and Pollatos (Critchley et al., 2004; Pollatos et al., 2007) and further modified and applied in fMRI studies of non-psychiatric and depressed participants by Wiebking et al. (2010, 2014, 2015). Briefly, the paradigm consists of three independent conditions (Supplementary Figure 2B). Each condition was presented 48 times in total in a pseudo-randomized order for 9–13 s each. Participants were instructed to direct their awareness to the external or the internal environment and count corresponding stimuli such as externally applied tones or the own heartbeat. Alternatively, a condition without an active task required no counting and served as baseline activity.

In more detail, participants were made familiar with the fMRI task before the scanning session. All participants were instructed by the same researcher (CW) following a standardized protocol. Each participant received the same instructions and all had the possibility to practice the paradigm on a computer outside the MRI room. For practice and scanning sessions the software Presentation (Neurobehavioral Systems) was used. The fMRI paradigm used simple visual stimuli to indicate one of the three condition types. All visual stimuli were dark colored pictures centralized on the same light background and had the same picture dimensions. In the scanner, an LCD projector was used to project the visual stimuli onto a screen visible through a mirror mounted on the headcoil. To indicate an IA condition, the task type indicator–a dark colored picture of a stylized heart–was presented on the same screen (jittered between 9–13 s). During these conditions individuals were asked to concentrate on their body and silently count their own heartbeat. Any kind of manipulation, such as holding their breath or evaluating their pulse at the radial artery, was not allowed.

During exteroceptive awareness (EA), participants were asked to focus on externally applied tones. As long as the task-type indicator–a dark colored picture of a musical note–was visible (jittered between 9–13 s) on the screen, study participants counted the number of externally applied tones. Afterwards, the number of counted heartbeats or tones was indicated on a rating scale (4 s). The indicator on the scale was moved by the subject to the labeled position representing the number of beats that they counted. Left and right button presses were used to move the indicator to the left and right side on the scale. This feedback component allowed the monitoring of the participant's attendance to the task.

Auditory stimuli were presented via the scanner loudspeaker. Tones were presented throughout the scanning sessions at an individually adapted volume to match the difficulty of both counting tasks. To ensure equivalent difficulty of both tasks, participants were instructed to adjust the volume of the tone to the same level of perception difficulty as that of counting their own heartbeat. This was done at the beginning of each of the four scanning sessions, i.e., with the scanner acquiring images to also account for scanner noise. Similar to the implementation of the rating scale, participants used right and left button presses to move an indicator on a rating scale corresponding to increase or decrease the volume. To illustrate, where the heartbeat counting was more difficult, an individual would lower the volume of the external tone in order to make this aspect of the task equally difficult to the heartbeat counting. This was explained to the participants before the scan. Being a standard part of each scanning session, participants also practiced this procedure outside the MRI room on a computer including speakers. In addition, the presentation frequency of the tones was adapted to correspond to each participant's heart-rate. The heart-rate was recorded using the Siemens Physiological Monitoring Unit (PMU) as described previously (Wiebking et al., 2014). In order to control for habituation effects, the individual onset time of each tone was jittered by 200 ms. Conditions with no particular task (Shulman et al., 2009) were indicated by a dark cross (9–13 s). Participants were instructed to disengage, reduce any cognitive work during these periods and maintain an undirected awareness, i.e., focusing neither on internal nor external stimuli. The total experiment consisted of four scanning sessions of 9.6 min each.

### MRI data acquisition and pre-processing

Functional echo planar images (EPI) were acquired using a 3-Tesla whole body MRI system (Siemens Trio, Erlangen, Germany). EPI with BOLD contrast were acquired using a body coil transmit and 8-channel receive headcoil. Thirty slices aligned at the AC-PC plane and covering the whole brain were acquired per volume. A total of 1160 volumes were collected over four scanning sessions per participant (FoV = 224 × 224 mm^2^; spatial resolution = 3.5 × 3.5 × 4 mm^3^; T_E_ = 30 ms; T_R_ = 2000 ms; flip angle = 80°). High resolution T_1_-weighted structural images were also acquired, using the following settings: MPRAGE; FoV = 256 × 256 mm^2^; spatial resolution = 1 × 1 × 2 mm^3^; T_E_ = 5 ms; T_R_ = 1650 ms.

Functional data were processed using FSL (http://www.fmrib.ox.ac.uk/fsl/) (Smith et al., 2004; Woolrich et al., 2009). Functional images were corrected for head movement (MCFLIRT) (Jenkinson et al., 2002) and motion outliers, brain-extracted (BET) (Smith, 2002), high-pass filtered with a 100 s cut-off, and smoothed with a 5 mm FWHM Gaussian kernel. Structural data were processed according to the FSL-VBM pipeline (Douaud et al., 2007) (http://fsl.fmrib.ox.ac.uk/fsl/fslwiki/FSLVBM). First, structural images were brain-extracted and gray matter-segmented before being registered to the MNI standard space using non-linear registration (Andersson et al., 2007). The resulting images were averaged and flipped along the x-axis to create a left-right symmetric, study-specific gray matter template. Next, all native gray matter images were non-linearly registered to this study-specific template and modulated to correct for local expansion (or contraction) due to the non-linear component of the spatial transformation. The modulated gray matter images were used to quantify the proportion of GM located in the regions of interest (see below).

Since structured noise still remains in the fMRI data after typical pre-processing steps, an independent component analysis (ICA) was applied to denoise the data and hence improve the sensitivity and specificity of the results. Using Probabilistic Independent Component Analysis (Beckmann and Smith, 2004) implemented in the MELODIC toolbox in FSL, a group ICA was performed on the pre-processed fMRI data. Components were visually inspected and classified as noise or signals of interest, according to a detailed description of an operationalized denoising procedure (Kelly et al., 2010). In particular, components were considered as noise when they showed a ring-like pattern in the periphery of the brain and tightly clustered areas in the frontal regions (McKeown et al., 1998), clusters with a location in the WM/cerebrospinal fluid or an association with blood vessels (Sui et al., 2009; Zou et al., 2009), spotted patterns diffusely spread over the brain, and time courses showing a saw-tooth pattern or spikes (McKeown et al., 1998). Individual timeseries from components that were identified as noise were removed from the original fMRI data through linear regression.

The statistical model for each participant involved the trial onsets and durations for the two conditions of interest (IA, EA) as well as parameters for motion outliers (FSL motion outliers) and movement (MCFLIRT). These were obtained during pre-processing steps and included as regressors to further minimize the effects of head movement. Specific condition effects for IA and EA (vs. implicit baseline) were tested by employing linear contrasts for each subject and different conditions using FEAT, Version 6.0 (Jenkinson et al., 2012). The resulting images were submitted to a second level random-effects analysis and subsequently two-sample unpaired *t*-tests were calculated on images obtained for each participant's volume set and different conditions using FLAME (details below).

### Higher-level statistical analyses (regression, MANOVA, group comparisons)

**Figure 1:**
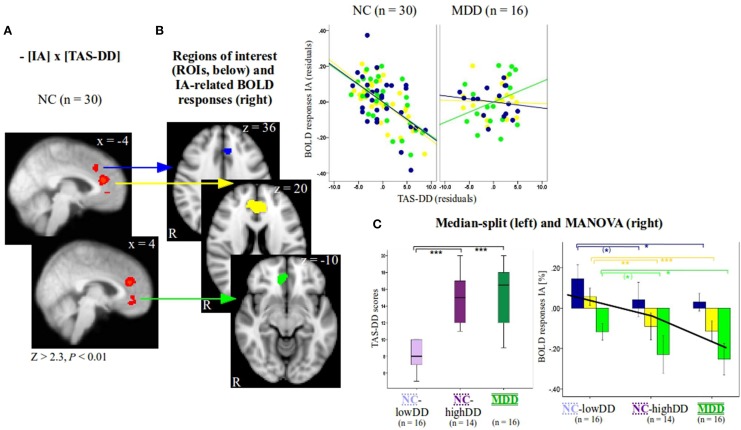
**(A)** Individual group slope for non-psychiatric controls (NC, *n* = 30, Z > 2.3, *P* < 0.01, x-coordinates are given in MNI space) as a function of TAS-DD scores. Group differences of IA-related activity between NC and patients with major depressive disorder (MDD) as a function of TAS-DD scores and individual results investigating MDD (*n* = 16, Z > 2.3, *P* < 0.05) can be seen in Supplementary Figure 1A. To visualize the negative relationship between IA-related activity and TAS-DD scores in NC, signal changes of the three medial-frontal regions (see **B**) were plotted against TAS-DD scores (values depict residuals since corrected for the amount of gray matter). **(B)** Three regions of interest (blue, yellow, and green ROI) were defined according to significant results as a function of TAS-DD scores seen in **(A)** (*n* = 30 NC). Z-coordinates are given in MNI space. **(C)** The group of NC (*n* = 30) was divided on the basis of a median-split into two subgroups, those with high (NC-highDD, *n* = 14) and those with low TAS-DD scores (NC-lowDD, *n* = 16). The three groups (NC-lowDD, NC-highDD, MDD) were defined as between-subjects factor when calculating MANOVA. TAS scores, BOLD responses of interoceptive and exteroceptive awareness (IA, EA), the amount of gray matter within the ROIs and age were defined as dependent within-subjects variables. *Post-hoc* tests (Bonferroni corrected) revealed no differences of TAS-DD scores between NC-highDD and MDD. Both groups differed significantly compared to NC-lowDD [see boxplots on the left hand side of **(C)** and Table 1B]. IA-related BOLD responses show a similar pattern. No differences occurred between high-alexithymic participants (NC-highDD and MDD), whilst both groups differed significantly to NC-lowDD (see bar diagram on right hand side of **(C)** and Table 1B for details; values show mean ± 95% confidence intervals). Please note that the color code of bars corresponds to the color of each ROI and to the colors used in Table 1B. The black line in bar diagram **(C)** shows mean IA-related BOLD responses of all participants per region, ranging from the blue to the yellow and green ROI (independent of group, see also Supplementary Table 2). ANOVA (Supplementary Table 2) revealed a significant effect of region. *** indicates *P* < 0.0001, ** indicates *P* < 0.001, * indicates *P* < 0.05, (*) indicates *P* < 0.1.

In the first step, the inference of interest was whether a linear relationship between neural activity (IA vs. implicit baseline or EA vs. implicit baseline) and TAS scores differed between groups (*n* = 16 MDD and *n* = 30 NC). The result indicated that the group difference significantly varied during IA in medial frontal regions as a function of TAS-DD (Supplementary Figure 1A). In order to obtain more information about the underlying factors, the calculation of individual group slopes was required (see results for NC in Figure 1A and for MDD in Supplementary Figure 1B). Whole-brain results were corrected using Z (Gaussianized T/F) statistic images. These were thresholded using clusters determined by Z > 2.3 and a cluster significance threshold of *P* < 0.01 in case of NC (Worsley, 2001). In case of MDD patients, a less conservative threshold of *P* < 0.05 was used, as the higher threshold was without any result.

Next, three functional regions of interest (ROIs, Figure 1B) were defined according to significant correlations as a function of TAS-DD scores seen in Figure 1A. Mean BOLD responses within these regions were calculated for each participant and each condition using Featquery. Values were entered into SPSS 17 (SPSS inc., Chicago, IL). For visualization purposes only, mean IA-related activity within significant voxels of activation was plotted against TAS-DD scores in NC and MDD (Figure 1B, colors in correspondence to color of each ROI, values depicting residuals since corrected for the amount of GM in each ROI).

The NC group was then divided into two subgroups according to TAS-DD scores using a median-split (boxplots in Figure 1C). This resulted in a group with high TAS-DD scorers (NC-highDD, *n* = 14) and a group with low TAS-DD scorers (NC-lowDD, *n* = 16). Thus, the continuous TAS scores were turned into a categorical variable. This approach can be used to investigate differences (or similarities) of neural activity during IA within the neurotypical participant group (comparing IA-related neural activity between high and low alexithymic individuals not suffering from any psychiatric disorder). Similarly, neural IA-related activity of participants showing the same range of self-reported TAS scores can be compared (high alexithymic non-psychiatric participants and depressed patients). In addition, equal sample sizes across the three groups ensure stable results independent of possibly skewed subject numbers.

IA or EA-related BOLD responses within ROIs showed no extreme outliers (farther than three interquartile ranges away from the first or third quartile) within the MDD group (*n* = 16) or the NC subgroups of high (*n* = 14) and low TAS-DD scorers (*n* = 16). To test differences of neural activity between these groups, a multivariate analysis of variance (MANOVA) was performed (Figure 1C, right side). The three participant groups (*n* = 16 MDD, *n* = 14 NC-highDD, *n* = 16 NC-lowDD) were defined as the between-subjects factor. TAS scores (DD, difficulties describing feelings; DI, difficulties identifying feelings; EO, externally oriented thinking), BOLD responses for each condition (IA and EA) and age were entered as dependent within-subjects variables. Since gray matter volumes have been associated with alexithymia (Borsci et al., 2009; Grabe et al., 2014), the amount of GM within each ROI was also included. Bonferroni correction was used for *post-hoc* testing in order to reduce type I errors (Table 1B and Figure 1C, colors correspond to color of each ROI). Differences between BHS scores were assessed by calculating univariate analysis of variance, as two BHS values were missing in the MDD group, which would lead to listwise exclusion of these individuals in the MANOVA. To investigate an effect of region in the three different ROIs independent of group, IA-related BOLD responses were pooled together (*n* = 46). An ANOVA was performed accordingly (between-subjects factor: three regions, dependent variable: IA-related BOLD responses of *n* = 46 participants, see Supplementary Table 2).

**Table 1:**
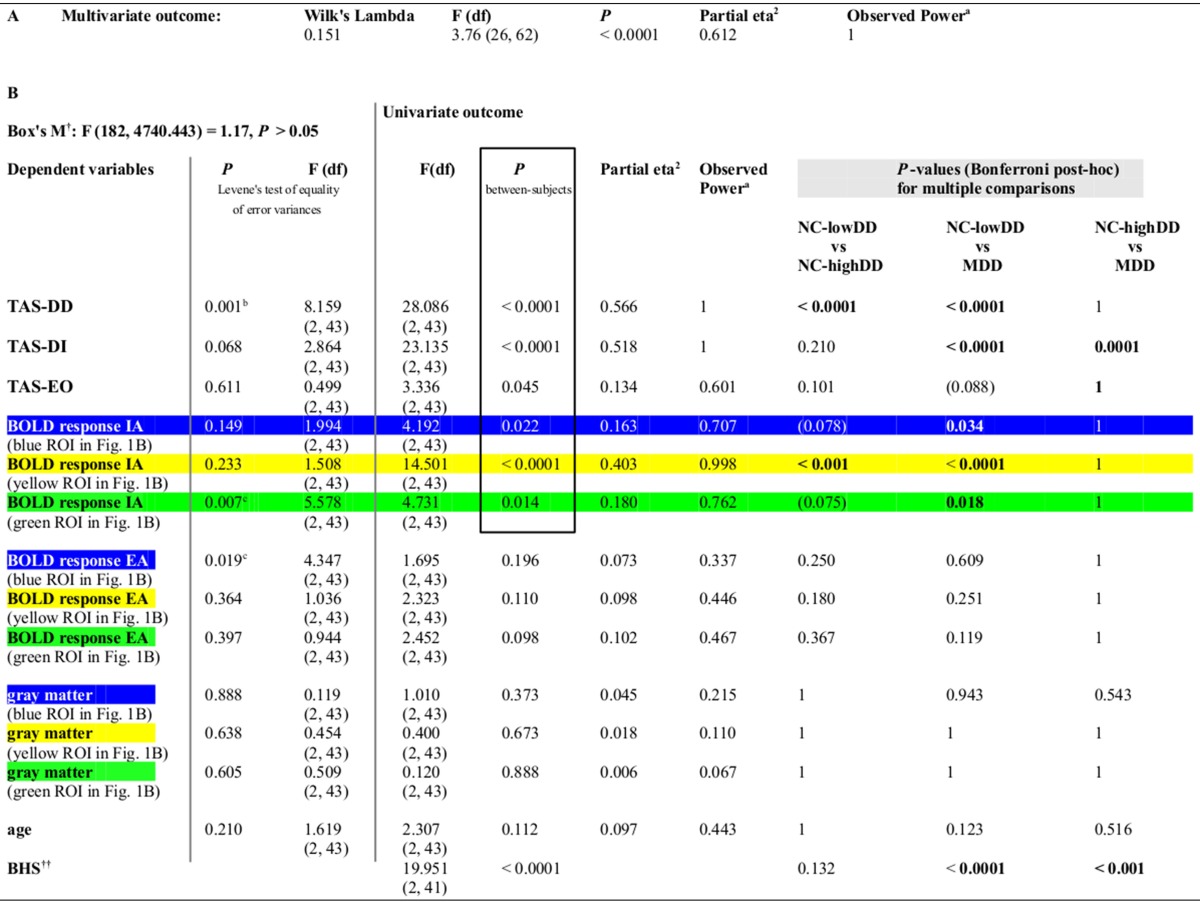
**(A) Using MANOVA, BOLD responses within the three regions of interest (defined according to Figures 1A,B) revealed a significant effect for group [*F*(hypothesis df: 26, error df: 62) = 3.76, *P* < 0.0001; Wilk's Lambda = 0.151, partial eta squared = 0.612]. (B) Significant between-subjects effects are highlighted by a box. *P*-values for pair-wise comparisons are based on Bonferroni corrections**.

*Groups include non-psychiatric controls (NC) with low TAS-DD scores (lowDD, n = 16), NC with high TAS-DD scores (highDD, n = 14) and patients with major depressive disorder (MDD, n = 16). Dependent variables include TAS scores (DD, difficulties describing feelings; DI, difficulties identifying feelings; EO, externally oriented thinking), BOLD responses during interoceptive and exteroceptive awareness (IA and EA) in the three different regions of interest (ROIs, according to Figure 1B), the amount of gray matter within each ROI and age. Please note that the color code corresponds to the color of each ROI seen in Figure 1B. The regions were defined according to significant correlations (n = 30 NC) as a function of TAS-DD scores (see Figure 1A and Supplementary Figure 1A). Differences between BHS scores were assessed by calculating univariate analysis of variance, as two values were missing in the MDD group, which would lead to listwise exclusion of these individuals in the MANOVA calculations*.

a alpha = 0.05

b *Unadjusted one-way ANOVA [F_*(2, 43)*_ = 6.069, P = 0.005] confirms MANOVA result as well as adjusted by Brown–Forsythe and Welch's statistics [Brown-Forsythe: F_*(2, 41.338)*_ = 6.013, P = 0. 005, Welch: F_*(2, 28.090)*_ = 6.275, P = 0.006]. For unequal variances the Games-Howell post-hoc results were checked with no differences compared to Bonferroni post-hoc results: NC-lowDD vs. NC-highDD: < 0.0001, NC-lowDD vs. MDD: < 0.0001, NC-highDD vs. MDD: 0.844*.

c *Unadjusted One-Way ANOVA shows no differences, Games-Howell post-hoc results reveal less strict results (for BOLD response IA: NC-lowDD vs. NC-highDD: 0.069. NC-lowDD vs. MDD: 0.010, NC-highDD vs. MDD: 0.915)*.

† *Box's M is not significant, providing assurance that the assumption of equality of covariance matrices is not violated*.

†† *Two missing values in MDD group. To avoid listwise exclusion of these data points in the MANOVA, the BHS results are based on univariate analysis of variance*.

**Figure 2:**
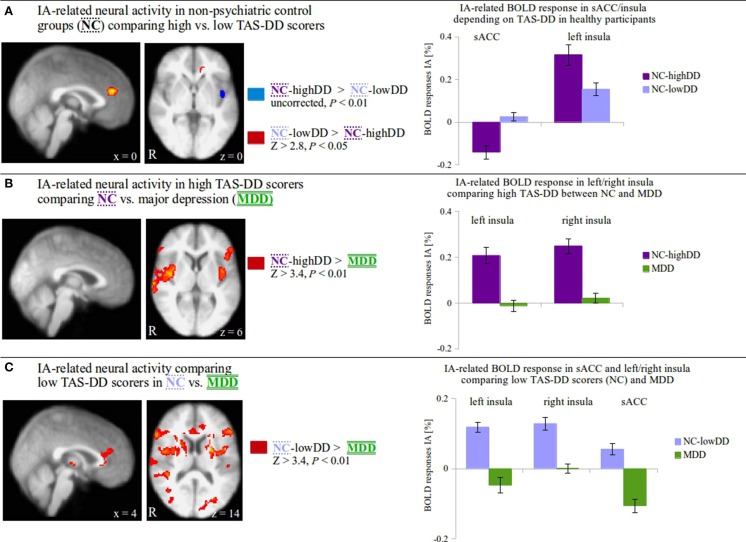
**(A)** Comparing IA-related neural activity between non-psychiatric controls (NC) with low (lowDD) and high TAS-DD scores (highDD) on a whole-brain level, alexithymic participants (NC-highDD) show increased activity in the left insula whilst NC-lowDD show increased activity in the supragenual anterior cingulate cortex (sACC; BA 24/32, coordinates in MNI space). A closer look at underlying neural activity during IA (bar diagrams show mean signal change ± SEM)**^†^** reveal positive BOLD responses in both groups in the insula with greater activity in NC-highDD (dark purple bars). In the sACC, NC-lowDD (light purple bars) reveal small positive BOLD responses, whilst the alexithymic group shows negative BOLD responses. **(B)** IA-related neural activity is compared between high alexithymic groups, i.e., non-psychiatric controls scoring high on the TAS-DD scale (NC-highDD) and patients suffering from major depressive disorder (MDD, coordinates in MNI space.). TAS-DD scores show no differences between these groups. On the whole-brain level, NC-highDD show significantly greater neural activity during IA in the insula compared to MDD. IA-related BOLD responses within the left and right insula (bar diagrams show mean signal change ± SEM)**^†^** illustrate that NC-highDD show positive BOLD responses (dark purple bars), whereas MDD show small responses (green bars). **(C)** When considering TAS-DD scores and psychiatric status, the comparison between NC-lowDD and MDD seems the most distinct. On the whole-brain level, NC-lowDD show significantly greater neural activity during IA in the left/right insula and the sACC (coordinates given in MNI space.). Calculating BOLD responses of each region (bar diagrams show mean signal change ± SEM)**^†^**, MDD patients show negative BOLD responses particularly in the left insula and sACC (green bars). NC-lowDD reveal positive IA-related activity particularly in the bilateral insula (light purple bars).**^†^** Bar diagrams for illustration purposes only. No additional statistical tests were performed on these values in order to avoid circularity.

The three groups (NC-highDD and NC-lowDD, which were defined according to self-reported TAS-DD scores, and MDD) revealed significant neural differences during IA in brain regions derived from negative correlations between TAS-DD and IA in a single group (NC). To further investigate the neural characteristics when directly comparing these *three* groups with each other, appropriate whole-brain comparisons were then performed (Figure 2, F-Test shown in Supplementary Figure 1B). In detail, IA-related activity was compared between non-psychiatric controls (NC) scoring high (NC-highDD) or low (NC-lowDD) on the TAS-DD scale (Figure 2A: NC-highDD vs. NC-lowDD, uncorrected, *P* < 0.01; NC-lowDD vs. NC-highDD, Z > 2.8, *P* < 0.05), between NC-highDD and patients with MDD (Figure 2B: NC-highDD vs. MDD, Z > 3.4, *P* < 0.01; no results for MDD vs. NC-highDD) and between NC-lowDD and MDD (Figure 2C: NC-lowDD vs. MDD, Z > 3.4, *P* < 0.01; no results for MDD vs. NC-lowDD). For visualization purposes, mean IA-related activity within significant voxels of activation was calculated and presented as bar diagrams (Figure 2, right side). Significant clusters of activation in Figures 2B,C that were located within insula/medial frontal cortex masks (as obtained from the Harvard-Oxford Cortical Structural Atlas included in FSL) were used for calculating mean IA and EA-related activity (see Supplementary Figures 2A,B).

## Results

### Comparing patients suffering from major depressive disorder (MDD, *n* = 16) to non-psychiatric controls (NC, *n* = 30) (Figure 1) and determination of NC subgroups

Firstly, we investigated whether a linear relationship between neural activity (IA vs. implicit baseline or EA vs. implicit baseline) and TAS scores differed between the *two* groups. 16 MDD patients were compared to the total group of 30 NC, i.e., this group was not classified according to their alexithymic state yet. Amongst the different TAS subscales, the relationship between IA-related activity and scores of the TAS-DD subscale was significantly different between these groups in medial-frontal regions (Supplementary Figure 1A). Individual group slopes were then calculated (see results for NC in Figure 1A and results for MDD in Supplementary Figure 1B), which showed negative correlations in those regions between TAS-DD and IA-related activity in NC, but not in MDD. Hence, the differences seen comparing MDD and NC were due to significant negative associations between TAS-DD and IA-related activity in the NC group. The results underline that alexithymia is linearly associated with decreasing neural activity during IA in medial-frontal regions in non-psychiatric control participants. For visualization purposes, mean IA-related BOLD responses within correlating clusters (please refer to regions in Figure 1B and Supplementary Table 1 for mean values) were plotted against TAS-DD scores (scatterplot in Figure 1B, values controlled for the amount of gray volume in respective ROIs). Additional statistical tests were not performed on these values in order to avoid circularity (e.g., Kriegeskorte et al., 2009).

Secondly, examining the categorical effect of TAS-DD scores on neural activity, the NC group was divided in a group with high TAS-DD scores (mean TAS-DD in NC-highDD: 14.57 ± 2.93) and a group with low TAS-DD scores (mean TAS-DD in NC-lowDD: 8.25 ± 1.57) using median-split. The MDD group had a mean TAS-DD score of 15.25 (± 3.75). Hence, MDD and NC-highDD were matched according to their TAS score (please refer to Table 1B for group comparisons and Supplementary Table 1 for mean values of the remaining TAS scales). TAS scores, BOLD responses during IA and EA within each ROI, gray matter and age were submitted as within-subjects variables to a MANOVA, whilst the *three* subject groups (NC-lowDD, NC-highDD, MDD) were defined as between-subjects factor. The MANOVA revealed a significant effect for group [*F*(hypothesis df: 26, error df: 62) = 3.76, *P* < 0.00001; Wilk's Lambda = 0.151, partial eta squared = 0.612, please refer to Table 1A]. As highlighted in Table 1B by a box, significant group effects occurred for each of the TAS subscales. In addition, BOLD responses during IA showed significant group effects for each of the *three* ROIs, which were defined according to significant correlations in NC (*n* = 30) as a function of TAS-DD scores seen in Figures 1A,B (please note that the color of each ROI corresponds to the color code used in Figure 1C and Table 1). Neural activity during EA or the amount of gray matter within each ROI showed no significant group effect (mean values in Supplementary Table 1).

Bonferroni *post-hoc* tests (Table 1B) revealed no significant TAS-DD differences between NC-highDD and MDD, whilst both groups differed significantly to NC-lowDD (as detailed in boxplots of Figure 1C, left). High alexithymic groups (i.e., NC-highDD as well as MDD) were therefore matched for TAS-DD scores. A similar pattern of group differences was observed in regard to IA-related activity. No differences occurred between NC-highDD and MDD, whilst both groups differed significantly to NC-lowDD. This pattern holds true for each of the *three* ROIs (see bar diagram in Figure 1C and Table 1B).

Combining IA-related BOLD responses of all participants (*n* = 46) and comparing them between these *three* ROIs, an ANOVA showed a significant effect for region (Supplementary Table 2, *P* < 0.0001). Each region showed significantly different IA-related BOLD responses compared to the remaining *two* ROIs (Supplementary Table 2). In more detail, the most dorsal region (blue ROI in Figure 1B, *z* = 36) exhibited positive BOLD responses across all *three* subject groups (Figure 1C: blue bars represent BOLD responses by group, black line represents mean value across all *n* = 46 participants, see also Supplementary Table 2). Compared to these positive BOLD responses seen in the dorsal ROI in each group, the sACC region (yellow ROI in Figure 1B, *z* = 20, closely corresponding to regions in Figures 2A,C) showed relatively diminished neural activity in each group (Figure 1C: yellow bars represent BOLD responses by group, black line represents mean value across all *n* = 46 participants, see also Supplementary Table 2). Compared to IA-related BOLD responses seen in the sACC, the most ventral region (green ROI in Figure 1B, *z* = −10) showed negative BOLD responses across all groups (Figure 1C: green bars represent BOLD responses by group, black line represents mean value across all *n* = 46 participants, see also Supplementary Table 2). In summary, mean IA-related BOLD responses of all study participants differ significantly between the *three* ROIs, whilst the most dorsal region (blue ROI) showed positive IA-related BOLD responses, the sACC (yellow ROI) showed comparatively lower neural activity and the most ventral region (green ROI) showed negative IA-related BOLD responses.

### Group comparisons between NC with high-alexithymic traits (NC-highDD, *n* = 14), low-alexithymic traits (NC-lowDD, *n* = 16) and MDD (*n* = 16) (Figure 2)

The MANOVA showed group differences during IA-related activity depending on TAS-DD scores. IA-related BOLD responses derived from regions that showed an association with TAS-DD scores in a group of non-psychiatric control participants, which was not subdivided according to their alexithymic trait. To investigate neural activity during IA between non-psychiatric control participants with high-alexithymic TAS-DD scores (NC-highDD), low-alexithymic TAS-DD scores (NC-lowDD) and depressed patients (having equally high TAS-DD scores), whole-brain comparisons between these three groups were performed (F-Test shown in Supplementary Figure 1B).

In a first step, IA-related activity on the whole-brain level was compared between the two non-psychiatric groups scoring high or low on the TAS-DD (Figure 2A, Z > 2.8, *P* < 0.05, red region). Non-psychiatric controls (NC) with low TAS-DD scores (NC-lowDD) showed increased IA-related activity in the supragenual anterior cingulate cortex (sACC; BA 24/32) when compared to NC with high TAS-DD scores (NC-highDD). Having a closer look at the underlying BOLD responses, NC-lowDD (light purple bars) revealed only small positive BOLD responses (bar diagrams in Figure 2A). The difference seen in the sACC was attributed to negative BOLD responses in the alexithymic group (dark purple bars, mean values in Supplementary Table 1). In contrast, IA-related activity was increased in NC-highDD in the left insula when compared to NC-lowDD (Figure 2A, uncorrected, *P* < 0.01, blue region). Plotting IA-related BOLD responses of this region depicted robust positive BOLD responses in both groups; however, alexithymic individuals showed the highest BOLD response in the left insula compared to low-alexithymic participants.

In a second step, IA-related activity on the whole-brain level was compared between high alexithymic groups, i.e., non-psychiatric controls scoring high on the TAS-DD scale and patients with MDD (Figure 2B, Z > 3.4, *P* < 0.01). Although TAS-DD scores did not differ between these groups (Figure 1C left, Table 1B), NC-highDD showed increased IA-related activity in the bilateral insula. Calculating BOLD responses within the left and right insula (bar diagrams in Figure 2B) showed low neural activity during IA in MDD (green bars), whereas NC-highDD showed again robust positive BOLD responses (dark purple bars, mean values in Supplementary Table 1). High alexithymic groups showed no differences in the sACC due to comparable negative BOLD responses in both groups [please compare negative BOLD response in the sACC in MDD (Figure 2C, green bar) to negative BOLD response in the sACC in NC-highDD (Figure 2A, dark purple bar)].

In a final step (Figure 2C), the two most distinct groups considering both TAS-DD scores and psychiatric status (NC-lowDD vs. MDD) were compared. On the whole-brain level, NC-lowDD showed greater neural activity during IA in the bilateral insula and the sACC (Z > 3.4, *P* < 0.01). Again, IA-related BOLD responses were calculated for each region and plotted by region (bar diagrams in Figure 2C). Whilst NC-lowDD showed positive BOLD responses particularly in the bilateral insula (light purple bars), MDD showed small and reduced neural activity particularly in the sACC (green bars, mean values in Supplementary Table 1). Please note that calculated signal changes seen in bar diagrams in Figures 2 A–C serve visualization purposes; no additional statistical tests were performed on these values in order to avoid circularity (Kriegeskorte et al., 2009).

## Discussion

This study forges a link between neural activity during IA and alexithymia in non-psychiatric controls and depressed individuals. To investigate IA, a well-established fMRI paradigm was used. Alexithymia was assessed using the TAS. In non-psychiatric controls (NC), TAS scores were negatively associated with IA-related activity in medial-frontal regions such as the supragenual anterior cingulate cortex (sACC; BA 24/32). Following a dimensional approach, where alexithymia is seen on a continuum and individuals may exhibit higher or lower degrees of alexithymia, high alexithymia in NC was associated with decreased activity in the sACC and increased activity in the insula when compared to low-alexithymic NC. Though having equally high TAS scores, high-alexithymic NC showed increased activity in the bilateral insula compared to MDD. Neural IA-activity in the sACC was similar between both high-alexithymic groups (i.e., MDD and NC-highDD).

### sACC: decreased neural activity in response to IA in high-alexithymic controls and major depressive disorder

When comparing neural activity during IA as a function of TAS-DD scores between non-psychiatric controls (NC, group consisting of *n* = 30 participants independent of TAS scores) and depressed participants (MDD, *n* = 16), group differences occurred in the medial-frontal cortex and specifically the sACC. In NC there is a clear negative relationship between IA-related neural activity and TAS-DD scores (Figure 1A), i.e., high alexithymia scores are associated with decreased neural activity in medial-frontal regions. Depressed patients showed no associations between TAS scores and neural activity in these areas.

The finding of decreased neural activity in non-psychiatric alexithymia is a well-documented neural response seen in many fMRI-studies (Berthoz et al., 2002; Moriguchi et al., 2007; Wingbermühle et al., 2012; Terasawa et al., 2013). Prior studies, however, used affective stimuli, such as emotional pictures of faces or body parts, to investigate neural correlates of alexithymia, implicating arousal and valence effects (e.g., van der Velde et al., 2013). The current study extends the existing literature by showing similar neural responses in non-psychiatric participants performing an IA task, i.e., independent of externally induced (visual-emotional or physical) stimulation. Participants merely shifted their awareness toward their own body (heartbeat counting) or toward the external environment (tone counting). Through this rather natural design of awareness switching it is possible to investigate the link between alexithymic traits on the subjective level and body awareness at the neural level. Both measures are positively associated on the subjective level of self-reports (e.g., Nyboe Jacobsen et al., 2006; Moriguchi and Komaki, 2013; Ernst et al., 2014). However, this is the first study linking alexithymia to neural activity during IA. Our first finding supports the assumption of decreasing neural activity in medial-frontal brain areas, as a function of alexithymic traits (see also bar diagram in Figure 1C).

**Figure 3:**
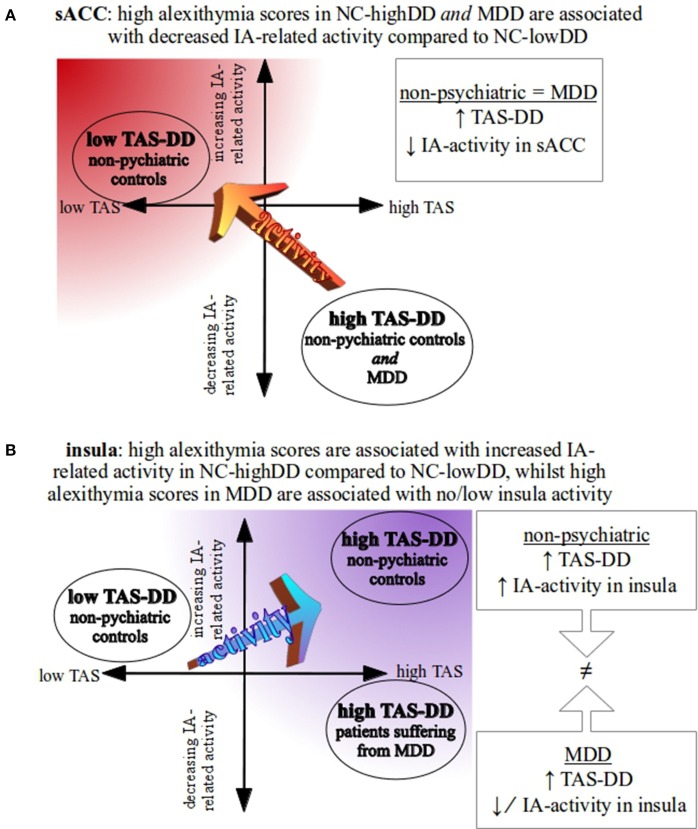
**Schematic overview of study-specific results. (A)** In the sACC, both high-alexithymic groups (NC-highDD and MDD) show a comparable pattern of negative IA-related activity in the supragenual anterior cingulate cortex (sACC, see also Figures 2A,C). Low TAS-DD scorers, however, show positive IA-related activity in the sACC (see also Figure 2C). **(B)** Comparing NC-highDD and NC-lowDD, participants with high alexithymia show greater positive BOLD responses in the insula during IA. As both groups consist of participants without psychopathological symptoms, this pattern may represent the typical association between alexithymia and IA-related activity in the insula. When comparing groups that show similar TAS-DD scores (NC-highDD and MDD), however, depressed patients show significantly lower IA-related activity in the insula (see also Figure 2B).

Further, we investigated BOLD differences/similarities between groups showing different/similar alexithymic status. For this, the subjective TAS-DD scores were used to subdivide the non-psychiatric group into high-alexithymic (NC-highDD) and low-alexithymic individuals (NC-lowDD). Instead of correlating self-reported alexithymia scores linearly with neural activity, as done previously, this step served as a dimensional approach to study different degrees of alexithymia. Thus, the continuous TAS scores were transformed into categorical variables (dichotomized using a median-split) leading to NC subgroups with high or low alexithymia scores. The advantage of this approach is twofold, as it can be used to investigate differences (or similarities) of IA-related neural activity (a) within a non-psychiatric control group comparing high/low alexithymic individuals (NC-highDD vs. NC-lowDD) and (b) between MDD and non-psychiatric individuals having the same range of TAS scores (NC-highDD vs. MDD). In addition, equal sample sizes across the three groups ensure stable results independent of potentially skewed subject numbers. When comparing healthy high and low TAS scorers on the whole-brain level, the results underline negative IA-related BOLD responses in the sACC in high-alexithymic compared to low-alexithymic NC participants (Figure 2A). This pattern may represent a common association between alexithymia and IA-related activity in the sACC, given the fact that both participant groups include individuals without psychopathological symptoms. This finding is accompanied by concomitantly increased IA-related BOLD responses in the insula, when comparing high vs. low-alexithymic NC participants, suggesting again this pattern (in combination with decreased sACC activity) may be a characteristic property of IA-related activity in alexithymia. The described neural differences in the sACC of healthy participants are in line with findings on the biochemical level, which show a positive relationship between levels of the inhibitory neurotransmitter GABA in the ACC and alexithymia. Though biochemical findings need to be interpreted with caution (Duncan et al., 2013), the features of GABA-mediated reductions in neural activity may account for decreased IA-related neural activity in alexithymia in this region (Ernst et al., 2014). When comparing IA-related activity between groups showing no differences in self-reported TAS scores (MDD and NC-highDD), decreased activity in the sACC in response to IA is similar between groups (please compare regional negative BOLD response in the sACC in MDD in Figure 2C to negative BOLD response in the sACC in NC-highDD in Figure 2A). This finding is suggestive of decreased sACC activity in high-alexithymic individuals independent of depression. Whole-brain results of neural activity during IA in the sACC are summarized in Figure 3A.

Furthermore, IA-related activity in the sACC appears to follow a regional dorsal-to-ventral gradient. As indicated by a black line in bar diagram Figure 1C (representing IA-related BOLD responses of all participants per region, ranging from the dorsal to the ventral ROI, independent of group, see Supplementary Table 2), IA-related activity decreased linearly from dorsal to ventral regions. This finding may shed light on the inconsistencies regarding ACC activity in alexithymia, since other studies have shown increasing ACC activity in alexithymia in response to emotional picture stimuli (Kano et al., 2007; Heinzel et al., 2010; Deng et al., 2013; Lemche et al., 2013; van der Velde et al., 2013). Besides possible differential effects of task type, task/arousal-load and valence-dependency in alexithymia (McRae et al., 2008; van der Velde et al., 2013), future studies should consider regional-specific activity patterns of the employed functional tasks. This could be realized by comparing BOLD responses along a dorsal-ventral gradient (or posterior-anterior, if applicable).

Briefly, within a sample representing the general population, high alexithymia was associated with increased insula and decreased sACC activity in response to IA compared to low alexithymia. Considering high-alexithymic depressed patients in comparison to high-alexithymic healthy participants, both groups showed comparable IA-related deactivation in the same sACC region. In addition, NC-highDD showed increased insula activity. Assuming a balance between sACC and insula activity as exemplified by the low-alexithymic group (low sACC activity and relatively higher positive insula responses during IA), the observed pattern in the high-alexithymic healthy group (decreased sACC and increased insula activity) could be interpreted as compensatory mechanisms to maintain a balanced system of IA-related neural activity between insula and sACC. In MDD, aberrant IA-related insula activity might account for alexithymic traits rather than sACC activity, as the latter shows no differentiation between high-alexithymic groups (MDD and NC-highDD).

### Insula: increased neural activity in response to IA in high-alexithymic controls, but aberrant in major depressive disorder

In concordance with fMRI studies investigating IA in the insula using a heartbeat counting task (Critchley et al., 2004; Wiebking et al., 2014), non-psychiatric participant groups (NC-highDD and NC-lowDD) showed robust positive IA-related activity in the insula (Figures 2A–C). Non-psychiatric alexithymic participants (NC-highDD) showed significantly higher neural activity in response to IA, when compared to NC-lowDD. As proposed by Kano and Moriguchi (Kano and Fukudo, 2013; Moriguchi and Komaki, 2013), heightened neural insula activity suggests amplified neural responses in alexithymic individuals in response to body-related stimuli. However, the present study is the first to show this neural pattern holds true during IA (as asked for by Kano and Fukudo, 2013), i.e., without externally applied emotional or physical stimulation. The current finding of increased IA-related activity in the insula of alexithymic individuals is supportive of theories suggesting increased awareness of somatosensory signals in alexithymia (e.g., De Gucht and Heiser, 2003; Kano et al., 2007; Ernst et al., 2014). However, heightened awareness of somatosensory signals in alexithymia does not necessarily correlate with objectified measures of sensitivity (Kano et al., 2007), specifically when dealing with psychiatric populations (Wiebking et al., 2010; Terhaar et al., 2012; Avery et al., 2014). The current study draws a link between alexithymia (that is typically accompanied by increased somatic awareness) and increased insula activity (that is related to somatic and emotional awareness, e.g., Craig, 2002, 2004; Critchley, 2005; Singer et al., 2009) in response to an IA task in non-psychiatric controls compared to MDD patients (showing the same degree of alexithymia). A direct link to somatosensory sensitivity, however, was not investigated in the current study.

Although speculative, increased IA-related insula activity in non-psychiatric alexithymia (NC-highDD) might mirror a compensatory mechanism within the scope of decreased sACC activity. In more detail, impaired emotional processing capacities, inherent in alexithymia (and mirrored by decreased activity in the sACC in MDD and NC-highDD), require a higher degree of body-related, interoceptive signals (mirrored by increased activity in the insula in healthy NC-highDD, but not in MDD) in order to sufficiently process emotional stimuli. This assumption is supported by close connections between insula and ACC on the anatomical level (Mesulam and Mufson, 1982; Nieuwenhuys et al., 2007; Moisset et al., 2010; in primates), as well as on the functional level showing connectivity (Taylor et al., 2009; Horn et al., 2010) and co-activation in response to various emotional paradigms (e.g., Singer et al., 2004; Bartels and Zeki, 2004). The insula can be described as a region integrating multimodal signals (Kelly et al., 2012; Farb et al., 2013) and both insula and ACC are crucial nodes of the salience network. Particularly the insula is seen as the integral hub of this network, which is assumed to mediate information flow across other neural networks, evaluate the most homeostatically relevant internal and external stimuli and, consequentially, guide behavioral responses to salient stimuli (Seeley et al., 2007; Craig, 2009; Menon and Uddin, 2010). As decreased sACC activity in response to IA is accompanied by upregulated insula activity in healthy alexithymics, it could be interpreted as a compensatory mechanism within the salience network found within neurotypical brain structures. This process is disrupted in MDD, which would mirror altered integrity of the salience network in alexithymia (as demonstrated for medial frontal areas of the default mode network by Liemburg et al., 2012). However, whether alterations within the salience network during IA are predominantly influenced by aberrant insula or sACC processing in alexithymia needs clarification in future analyses using additional methods, such as connectivity analyses or psychophysiological interactions.

In a second comparison, neural activity during IA was compared between high TAS-DD scorers (NC-highDD and MDD, Figure 2B). TAS-DD scores between both groups did not differ, but non-psychiatric controls showed increased IA-related activity in the bilateral insula compared to the MDD group. A similar pattern can be seen when comparing NC-lowDD to MDD (Figure 2C). Again, the non-psychiatric group (NC-lowDD) showed robust positive BOLD responses, but depressed participants revealed only low or negative BOLD responses. In contrast to the sACC, the insula was affected in both contrasts involving MDD. Research investigating IA-related neural activity in MDD observed impaired neural activity in the insula in depressed individuals (Avery et al., 2014; Wiebking et al., 2015), suggesting that the differences observed in the current study might be related to overall reduced insula activity independent of alexithymic traits, and hence account for general deficits of physiological processing capacities in MDD. Reduced functional connectivity between insula and sACC in psychopathology within the salience network (Menon, 2011) supports this assumption. Whole-brain results of IA-related neural activity in the insula are summarized in Figure 3B.

Taken together, the current study is the first investigating neural activity in the insula during IA (heartbeat awareness) in non-psychiatric participants scoring high or low on the TAS-DD subscale and patients suffering from MDD scoring equally high on the TAS-DD scale. Higher positive BOLD-responses in response to IA were seen in non-psychiatric alexithymic individuals (NC-highDD), but not in the MDD group with equally high TAS-DD scores. This finding suggests an upregulated insula activity in response to interoceptive, body-related awareness in non-psychiatric alexithymia and may be seen as a compensatory mechanism within the scope of decreased sACC activity. As depressed patients show similarly decreased BOLD responses in the sACC, but unbalanced insula activity, these processes seem to be disrupted in depression.

### Limitations

Patients with MDD participating in the current study were under medication. Thus, a medication effect in comparison to the non-psychiatric group cannot be fully excluded. Future studies should control for these influences more rigorously and include unmedicated patients as well. Moreover, it must be pointed out that the investigation of participants' sensitivity to internal processes, i.e., the distinction between good and poor heartbeat perceivers (Pollatos and Schandry, 2004; Herbert et al., 2011) and subsequent whole-brain comparisons, was not targeted in this study. Whilst the performance of heartbeat perception on the behavioral level is inconclusive in depression (Dunn et al., 2007; Terhaar et al., 2012), possible differences in accuracy might be related to cognitive impairments and/or reduced heartbeat evoked potentials in MDD (Terhaar et al., 2012) and thus resemble confounding factors for such analyses. Specifically the impaired heartbeat evoked potentials suggest that the neural activity underlying the interoceptive stimulus response might be altered in MDD *per se* (Avery et al., 2014). The central interest of the current study was to investigate neural activity during awareness directed toward the internal (heartbeat) or external environment (tones), independent of individual task performance or sensitivity (please refer to Garfinkel et al., 2014 distinguishing interoceptive accuracy from interoceptive awareness). Further research carefully considering those factors and including long counting periods without auditory stimulation is needed to clarify the exact relationship between heartbeat counting performance/accuracy and neural activity during heartbeat counting in health and depression. Finally, methodological concerns could be raised with respect to the subjective measure used (TAS-20). Results of this self-report questionnaire may be biased by self-presentation concerns and social desirability. Controlling for these factors, future studies investigating alexithymia should additionally use observer-based scales, such as the Toronto Structured Interview for Alexithymia (TSIA, Bagby et al., 2006), which show high correlations with TAS-20.

## Conclusions

In contrast to neuroimaging studies on emotional processes of alexithymia, often done through emotional picture viewing, the current study focuses on the relationship between neural activity during awareness of body-related, interoceptive stimuli (heartbeat) in combination with subjective scores of alexithymia in depressed and non-psychiatric participants. The results of the current fMRI study show that alexithymia is associated with altered IA-related activity in the sACC (significantly decreased activity in NC-highDD and MDD) and insula (significantly increased activity in NC-highDD, but not MDD). In the context of decreased interoceptive activity in the sACC, increased insula activity in response to interoceptive signals could be a compensatory mechanism within the salience network in non-psychiatric alexithymia, which is decoupled in MDD.

## Author contributions

CW and GN conceptualized research; CW developed paradigm, acquired data, analyzed data and wrote the paper.

### Conflict of interest statement

The authors declare that the research was conducted in the absence of any commercial or financial relationships that could be construed as a potential conflict of interest.
