# Supplementary material for: Neural activity during interoceptive awareness and its associations with alexithymia—An fMRI study in major depressive disorder and non-psychiatric controls
Source: Front Psychol. 2015 May 27;6:589. doi: 10.3389/fpsyg.2015.00589 (PMC4444750; doi:10.3389/fpsyg.2015.00589)
Supplement: Supplementary file 1 [file Table1.PDF]

Suppl. table 1

	NC total group	MDD	TAS-DD median-split	TAS-DD median-split
	NC (n = 30)	MDD (n = 16)	NC-highDD (n = 14)	NC-lowDD (n = 16)
	mean ± SD	mean ± SD	mean ± SD	mean ± SD
**TAS-DD**	11.20 ± 3.93	15.25 ± 3.75	14.57 ± 2.93	8.25 ± 1.57
**TAS-DI**	11.87 ± 3.78	20.19 ± 4.97	13.36 ± 4.40	10.56 ± 2.63
**TAS-EO**	17.90 ± 4.30	19.69 ± 4.63	19.71 ± 3.95	16.31 ± 4.06
**Age**	33.73 ± 11.62	41.19 ± 11.78	35.21 ± 13.47	32.44 ± 9.99
**Education**	16.05 ± 2.42	15.72 ± 2.88	15.53 ± 2.34	16.50 ± 2.47
**BHS**[a]	4.60 ± 3.91	12.29 ± 4.60 †	6.21 ± 4.23	3.19 ± 3.08
**BOLD responses Figure 1**				
**BOLD response IA** (blue ROI in Fig. 1B)		0.031 ± 0.082	0.042 ± 0.149	0.145 ± 0.131
**BOLD response IA** (yellow ROI in Fig. 1B)		-0.114 ± 0.093	-0.090 ± 0.116	0.057 ± 0.079
**BOLD response IA** (green ROI in Fig. 1B)		-0.253 ± 0.148	-0.230 ± 0.161	-0.117 ± 0.078
**BOLD response EA** (blue ROI in Fig. 1B)		0.020 ± 0.053	-0.002 ± 0.146	0.072 ± 0.130
**BOLD response EA** (yellow ROI in Fig. 1B)		-0.040 ± 0.082	-0.048 ± 0.117	0.025 ± 0.109
**BOLD response EA** (green ROI in Fig. 1B)		-0.150 ± 0.155	-0.127 ± 0.137	-0.048 ± 0.112
**BOLD responses Figure 2**				
**BOLD response IA in left insula** (Fig. 2A)		0.026 ± 0.135	0.315 ± 0.174	0.155 ± 0.116
**BOLD response IA in sACC** (Fig. 2A)		-0.144 ± 0.096	-0.141 ± 0.119	0.026 ± 0.078
**BOLD response IA in left insula** (Figure 2B)		-0.012 ± 0.101	0.208 ± 0.130	0.122 ± 0.077
**BOLD response IA in right insula** (Fig. 2B)		0.022 ± 0.084	0.248 ± 0.124	0.176 ± 0.120
**BOLD response IA in left insula** (Fig. 2C)		-0.047 ± 0.090	0.010 ± 0.128	0.119 ± 0.057
**BOLD response IA in right insula** (Fig. 2C)		0.001 ± 0.054	0.132 ± 0.101	0.128 ± 0.074
**BOLD response IA in sACC**(Fig. 2C)		-0.106 ± 0.076	-0.059 ± 0.103	0.055 ± 0.066
**BOLD response EA in left insula** (Fig. 2A)		-0.021 ± 0.086	0.121 ± 0.086	0.059 ± 0.132
**BOLD response EA in sACC** (Fig. 2A)		-0.055 ± 0.095	-0.062 ± 0.124	0.013 ± 0.119
**BOLD response EA in left insula** (Fig. 2B)		-0.029 ± 0.043	0.069 ± 0.059	0.041 ± 0.078
**BOLD response EA in right insula** (Fig. 2B)		-0.016 ± 0.064	0.073 ± 0.062	0.065 ± 0.082
**BOLD response EA in left insula** (Fig. 2C)		-0.017 ± 0.036	0.022 ± 0.069	0.048 ± 0.057
**BOLD response EA in right insula** (Fig. 2C)		0.003 ± 0.040	0.028 ± 0.055	0.044 ± 0.061
**BOLD response EA in sACC** (Fig. 2C)		-0.049 ± 0.055	-0.042 ± 0.090	0.016 ± 0.093

[a] BHS-scores range from 0-20

† Two missing values in MDD group.

Suppl. table 2

**region 1 (n = 46)**†	**0.074 ± 0.13 (mean ± SD)**
**region 2 (n = 46)**†	**-0.047 ± 0.12 (mean ± SD)**
**region 3 (n = 46)**†	**-0.200 ± 0.14 (mean ± SD)**

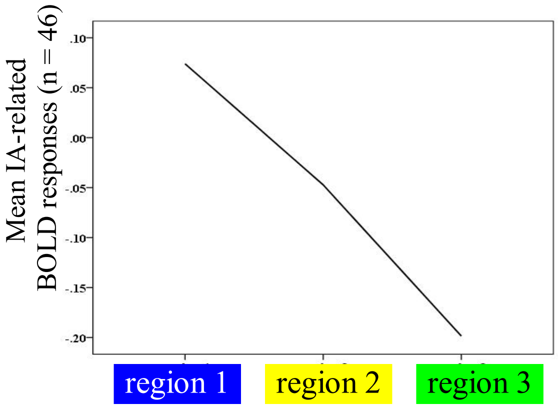

**Dependent variable**	**F (df)**	***P*** between-subjects	***P*-values [a] for multiple comparisons** region 1 vs region 2	region 1 vs region 3	region 2 vs region 3
**ANOVA**					
**BOLD response IA**†	48.97 (2, 135)	< 0.0001	**< 0.0001**	**< 0.0001**	**< 0.0001**

[a] *P*-values are given in **bold** for significant contrasts ($P < 0.05$) based on Bonferroni post-hoc tests.

† To establish an effect of region independent of group, IA-related BOLD responses include all particiapants independent of group, i.e., 30 NC + 16 MDD = 46. (Degrees of freedom (df) mirror regions and 46 subjects * 3 regions).
