# Supplementary material for: Neural activity during interoceptive awareness and its associations with alexithymia—An fMRI study in major depressive disorder and non-psychiatric controls
Source: Front Psychol. 2015 May 27;6:589. doi: 10.3389/fpsyg.2015.00589 (PMC4444750; doi:10.3389/fpsyg.2015.00589)
Supplement: Supplementary file 2 [file Image1.PDF]

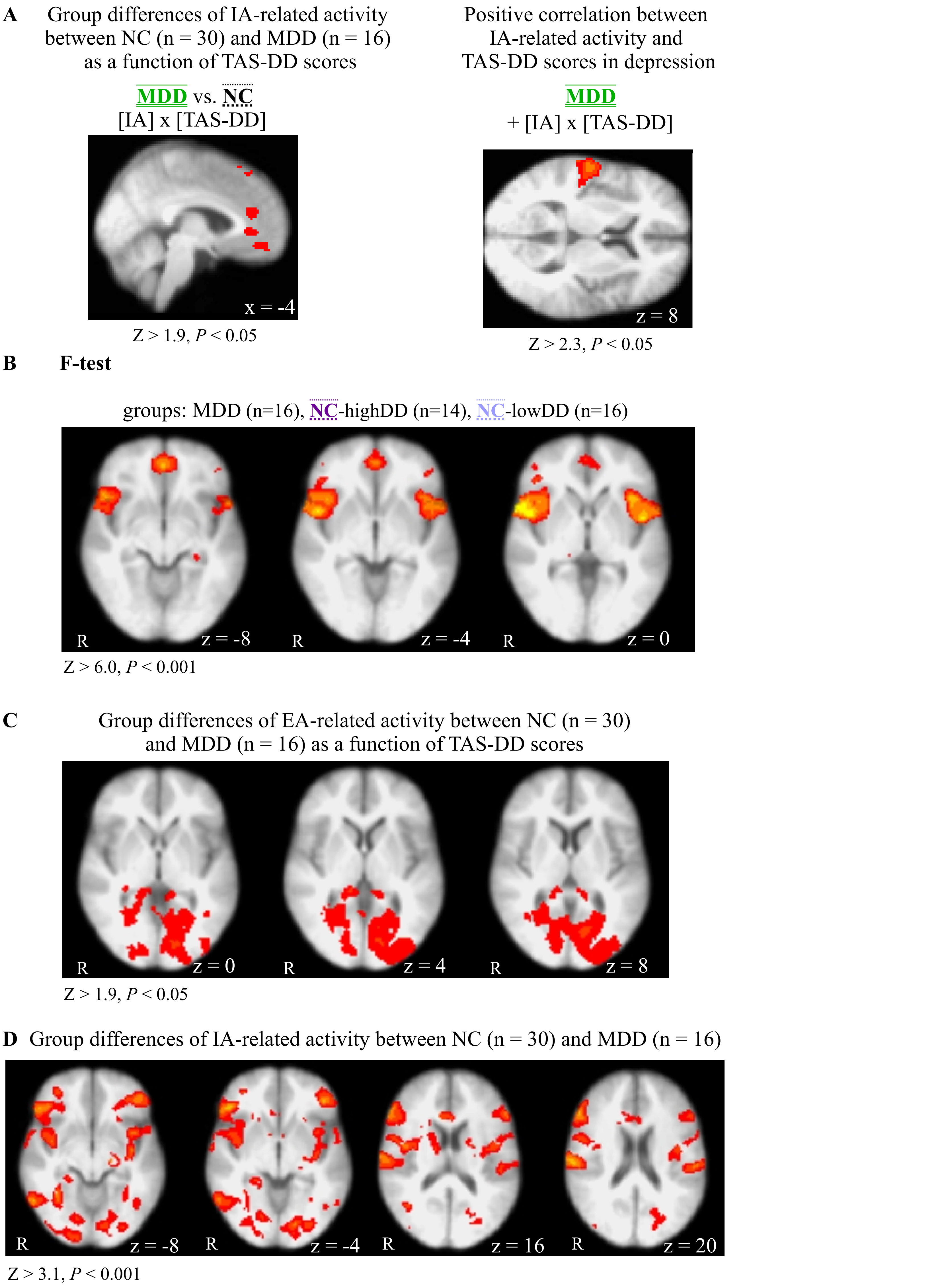

Suppl. figure 1

A Significant group differences of IA-related activity between non-psychiatric controls (NC, n = 30) and patients suffering from major depressive disorder (MDD, n = 16) as a function of TAS-DD scores in medial frontal brain regions (left side, Z > 1.9, *P* < 0.05). Positive correlation between IA-related activity and TAS-DD scores in the group of depressed participants (right side, n = 16, Z > 2.3, *P* < 0.05). Coordinates are given in MNI space.

B F-test showing that the three group averages account for significant BOLD effects during IA (n = 14 NC-highDD, n = 16 NC-lowDD, n = 16 MDD, Z > 6.0, *P* < 0.001). As the F-test is significant, individual t-tests are used to determine the direction of effect, which are shown in Figure 2. Coordinates are given in MNI space.

C Significant group differences of EA-related activity between non-psychiatric controls (NC, n = 30) and patients with major depressive disorder (MDD, n = 16) as a function of TAS-DD scores in regions of the visual cortex (threshold of Z > 1.9 and *P* < 0.05 equals threshold applied in Suppl. figure 1A). Coordinates are given in MNI space.

D Significant group differences of IA-related activity between non-psychiatric controls (NC, n = 30) and patients with major depressive disorder (MDD, n = 16, Z > 3.1, *P* < 0.001). Coordinates are given in MNI space.
