# Supplementary figures and images for: Neural activity during interoceptive awareness and its associations with alexithymia—An fMRI study in major depressive disorder and non-psychiatric controls
Source: Front Psychol. 2015 May 27;6:589. doi: 10.3389/fpsyg.2015.00589 (PMC4444750; doi:10.3389/fpsyg.2015.00589)

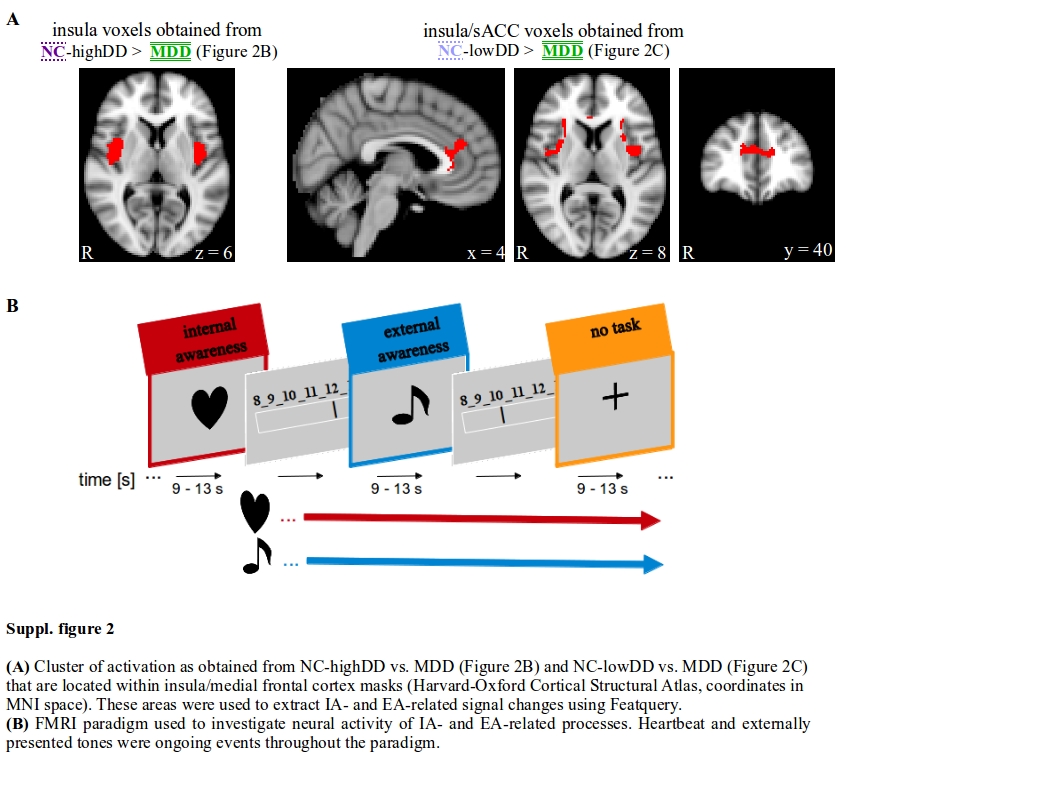

Supplement: Supplementary file 3 [file Image2.JPEG]
